# A Case of Malignant Myxofibrosarcoma With Hypoglycemia Attacks

**DOI:** 10.7759/cureus.38387

**Published:** 2023-05-01

**Authors:** Muhammed Yusuf Afacan, Nuri Ayoglu, Mahmut Kursat Ozsahin, Huseyin Botanlioglu

**Affiliations:** 1 Department of Orthopaedics and Traumatology, Istanbul University-Cerrahpasa, Cerrahpasa Medical Faculty, Istanbul, TUR

**Keywords:** insuline-like growth factors, blood glucose, myofibroblastic tumor, targeted therapy, insulin, postoperative radiotherapy, wide surgical resection, tru-cut biopsy, hypoglycemia attacks, malignant myxofibrosarcoma

## Abstract

Myxofibrosarcoma is a malignant mesenchymal tumor and a fibroblastic sarcoma of the elderly. Myxofibrosarcoma can be low-grade or high-grade depending on the cell characteristics. Wide surgical resection with or without radiotherapy and chemotherapy is the basis of its treatment. Sometimes, tumor cells secrete insulin or insulin-like substances and cause hypoglycemia attacks. Here, we intend to demonstrate the role of early surgery to end hypoglycemia attacks and prevent recurrence and metastases. We also intend to show the insufficiency of tru-cut biopsy to distinguish between low- and high-grade myxofibrosarcoma. An 82-year-old male patient visited our clinic with a rapidly growing giant mass in the left retroscapular area and suffered from hypoglycemic attacks several times a day. After imaging and initial biopsy, the tumor grade was indeterminate on histopathological examination; hence, the mass was removed surgically. The pathological examination resulted in high-grade myxofibrosarcoma whereas the initial biopsy could not elaborate on the grade. The hypoglycemia attacks ceased after the surgery. Adjuvant local radiotherapy at a total dose of 60 Gy was administered in 30 fractions to the surgery area with no complications after the surgery. No new mass, recurrence, or hypoglycemia attack was detected in the three-year follow-up. In conclusion, hypoglycemia attacks may be a marker of malignant tumor presence and may be a clue at the beginning and in the follow-up period both for recurrence and the aggressiveness of the tumoral mass. Because a biopsy may show the diagnosis but not the grade of the tumor, early surgical intervention is needed.

## Introduction

Although myxofibrosarcoma, known as myxoid malignant fibrous histiocytoma, is a rare soft tissue spindle cell malignant mesenchymal tumor, it is the most common fibroblastic sarcoma in older patients [1]. Myxofibrosarcoma is characterized by malignant fibroblasts dispersed within the myxoid matrix, and it is most prevalent in men aged 60 to 80 [2]. Myxofibrosarcomas may be low-grade or high-grade and may cause local invasion or whole-body metastasis. High-quality magnetic resonance imaging (MRI) guidance is crucial in preoperative planning. Wide surgical resection with a 2 cm surgical margin is the basic principle of treatment. Complex vascular and plastic surgery reconstructive techniques may be needed during surgery [3]. Insulin and insulin-like substances secreted by tumor cells can cause hypoglycemia attacks in patients with paraneoplastic syndrome. Despite the presence of paraneoplastic hypoglycemia in diverse soft-tissue sarcomas associated with increased insulin-like growth factor 2 (IGF-2), defined as non-islet cell tumor hypoglycemia, paraneoplastic hypoglycemia associated with myxofibrosarcoma was reported rarely [4]. One case of retroperitoneal myxofibrosarcoma revealed secretion of IGF-2 by radioimmunoassay and was associated with paraneoplastic hypoglycemia [4]. Our aim in this study is to emphasize the role of early surgery in preventing local invasion and widespread distant metastases, correlating wide resection with ending hypoglycemic attacks by preventing the further production of IGF-2 and insufficiency of tru-cut biopsy to distinguish between low- and high-grade myxofibrosarcoma in a patient diagnosed with myxofibrosarcoma with an aggressive growth pattern and a mass that reached giant dimensions within a short time.

## Case presentation

**Figure 1:**
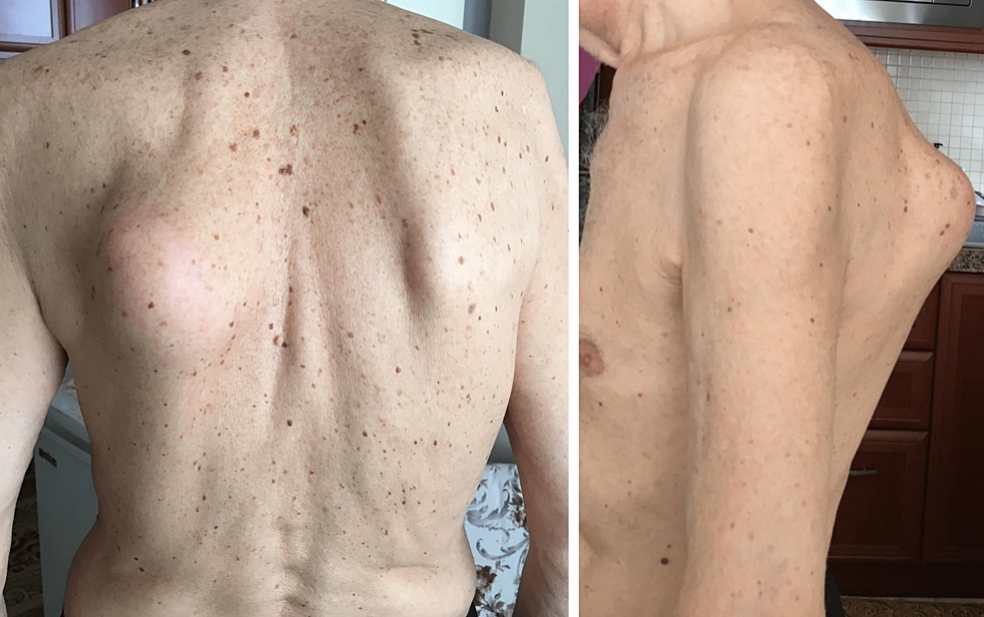
The giant mass lesion on the left retroscapular region.

An 82-year-old male patient visited our clinic with a painful mass presenting a high growth tendency in the left retroscapular area. The mass first appeared five months ago and showed rapid growth in the past two months (Figure 1).

**Figure 2:**
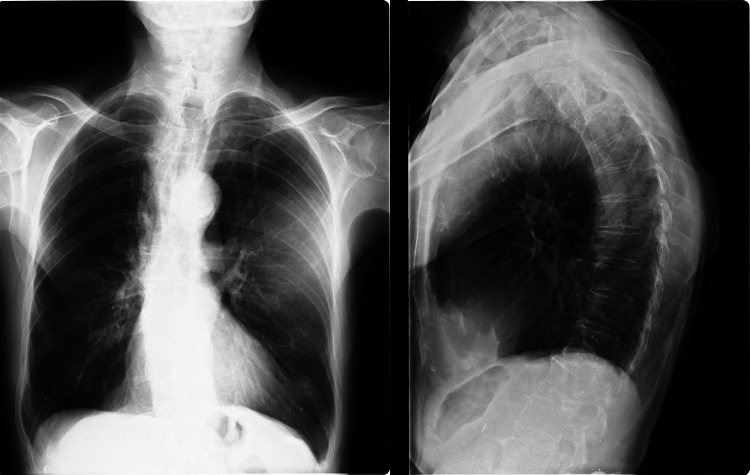
The space-occupying lesion on the posteroanterior and lateral chest radiograph.

The patient suffered from hypoglycemic attacks several times a day as his glucose level ranged between 45 and 55 mg/dL. The patient’s potassium levels also ranged between 2.5 and 3.5 mEq/L, indicating mild hypokalemia during the attacks. A space-occupying lesion was detected on the posteroanterior and lateral chest radiograph (Figure 2).

**Figure 3:**
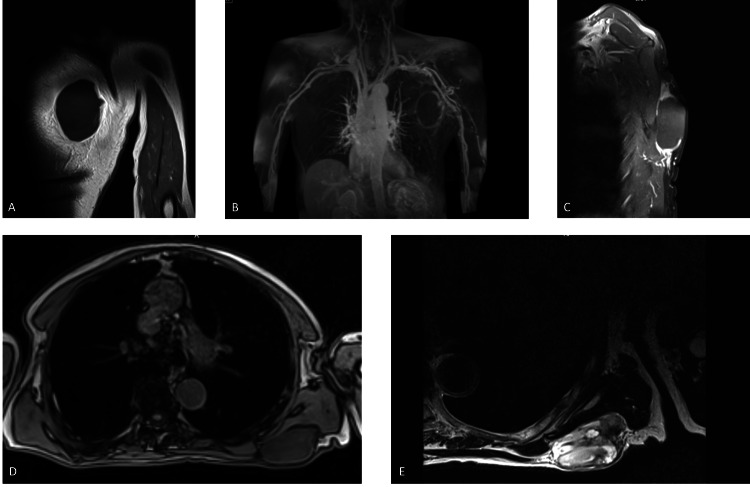
Intravenous contrast-enhanced magnetic resonance imaging. A: T1-weighted turbo spin-echo coronal section of the intravenous contrast-enhanced magnetic resonance imaging performed for the patient’s mass. B: T1-weighted fat suppression coronal section of the intravenous contrast-enhanced magnetic resonance imaging performed for the patient’s mass. C: T1-weighted turbo spin-echo fat suppression sagittal section of the intravenous contrast-enhanced magnetic resonance imaging performed for the patient’s mass. D: T1-weighted transverse section of the intravenous contrast-enhanced magnetic resonance imaging performed for the patient’s mass. E: T2-weighted transverse section of the intravenous contrast-enhanced magnetic resonance imaging performed for the patient’s mass.

On intravenous contrast-enhanced MRI performed for the patient’s mass, the mass lesion measured approximately 9.5 × 5.5 × 7 cm on the surface of the left infraspinatus muscle (Figure 3).

**Figure 4:**
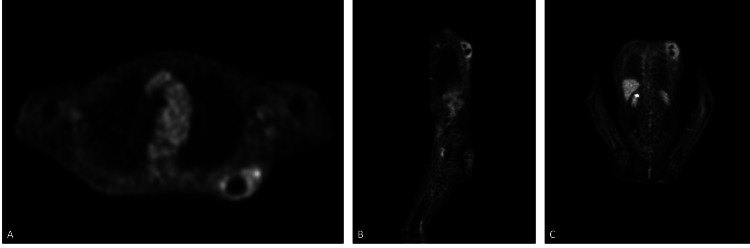
Positron emission tomography/computed tomography imaging. A: Transverse section of the F18-fluorodeoxyglucose positron emission tomography/computed tomography imaging revealing a primary tumoral mass lesion in the dorsal region accompanied by hypometabolic areas due to possible necrosis in central regions. B: Saggital section of the F18-fluorodeoxyglucose positron emission tomography/computed tomography imaging revealing a primary tumoral mass lesion in the dorsal region accompanied by hypometabolic areas due to possible necrosis in central regions. C: Coronal section of the F18-fluorodeoxyglucose positron emission tomography/computed tomography imaging revealing a primary tumoral mass lesion in the dorsal region accompanied by hypometabolic areas due to possible necrosis in central regions.

F18-fluorodeoxyglucose positron emission tomography/computed tomography examination was performed, revealing a primary tumoral mass lesion in the dorsal region without metastasis, extending to the subcutaneous tissue located in the middle part of the scapula on the left but not showing significant invasion in the bone structure, accompanied by hypometabolic areas due to possible necrosis in central regions (maximum standardized uptake value: 4.4) (Figure 4).

A tru-cut biopsy was performed for histopathological examination in the presence of interventional radiology. The biopsy showed a low-intermediate spindle cell malignant mesenchymal tumor with a focal myxoid component. Because of the superficial mass, patient’s age, pleomorphic cells in between, myxoid component and vascular features, and CD34 positivity, the pathologists deduced myxofibrosarcoma exhibiting fibroblastic-myofibroblastic differentiation. Because high-grade myxofibrosarcoma could not be excluded after biopsy, an excision of the mass was recommended, considering the surgical margins.

Surgery was planned for the patient diagnosed with myxofibrosarcoma measuring approximately 9.5 × 5.5 × 7 cm at the posterior of the left scapula, with the indication of mass excisional biopsy.

**Figure 5:**
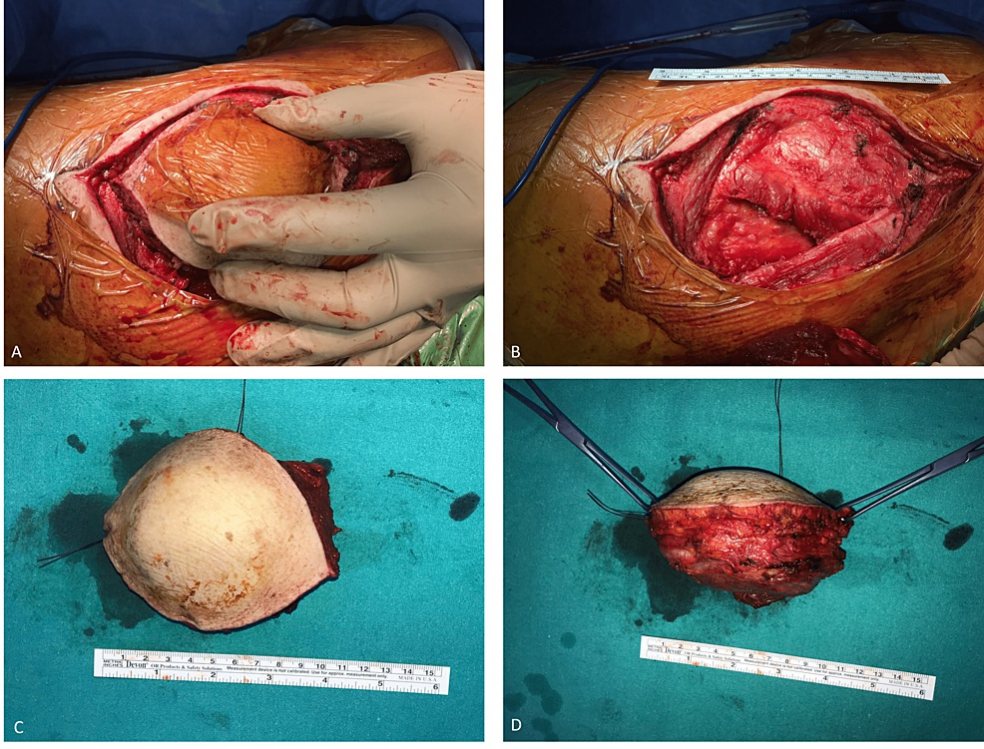
The incision and removed mass. A: An approximately 15 cm incision over the mass in the form of a fish mouth before removal of the mass with the skin. B: The body space remaining after removal of the mass with wide resection involving the skin. C: The anterior view of the removed mass lesion with the skin after wide resection. D: The lateral view of the removed mass lesion with the skin after wide resection.

The mass boundaries were marked at the posterior aspect of the scapula. Starting from the proximal to the distal and medial, an approximately 15 cm incision was made over the mass in the form of a fish mouth to encompass the skin. The mass was removed totally (en bloc) with the capsule. The excised material was sent to pathology for examination (Figure 5).

**Figure 6:**
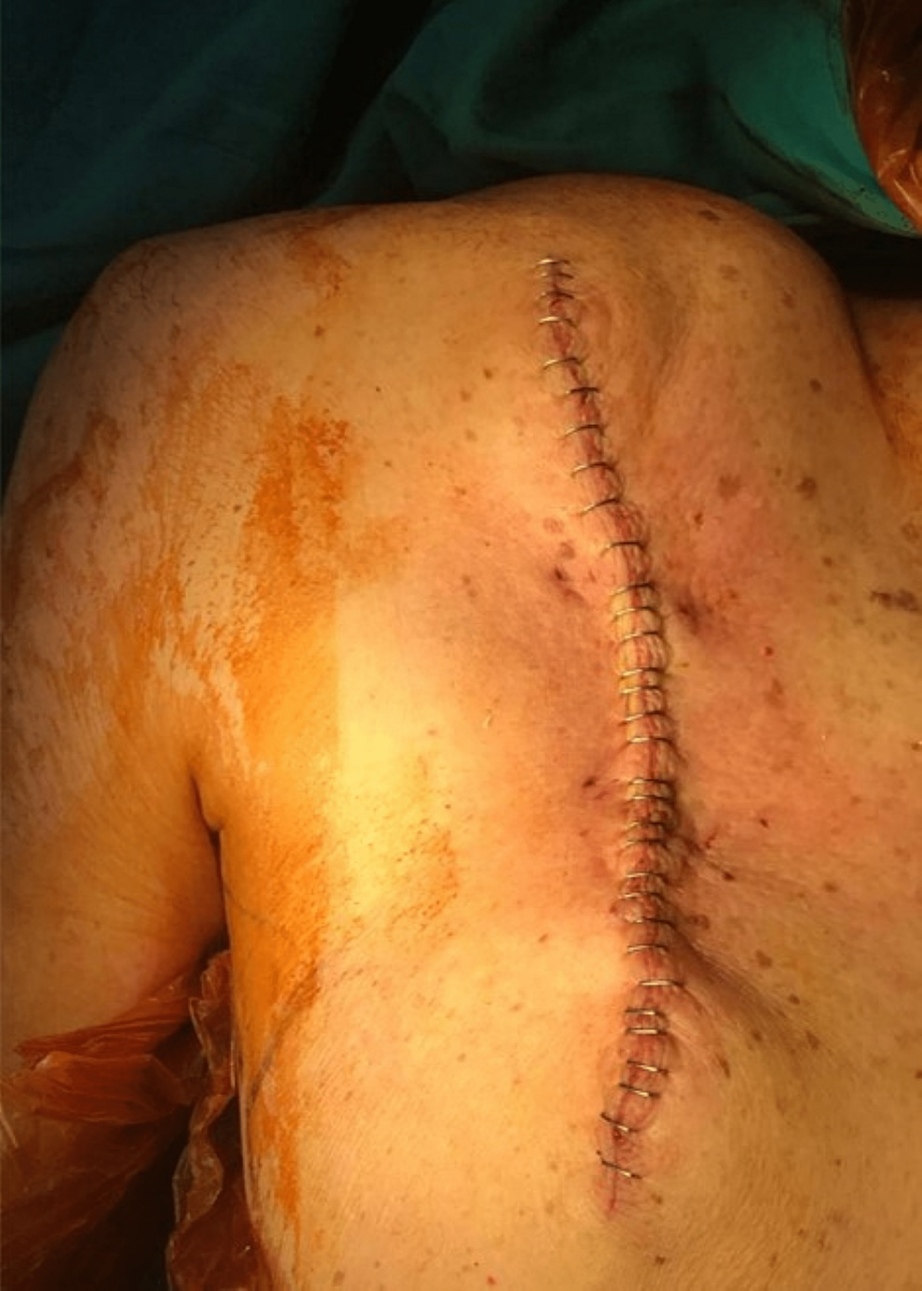
The dorsal region of the patient after the surgery. The surgical layers were closed over a drain in accordance with their anatomy at the end of the surgical procedure after the frozen section had resulted in a clear surgical margin

The frozen section resulted in a clear surgical margin. The surgical layers were closed over a drain in accordance with their anatomy (Figure 6).

The pathological diagnosis of the patient was high-grade myxofibrosarcoma. A postoperative shoulder sling was applied and the patient’s movements were restricted for seven days to avoid any problems in the incision line in the back area. Passive range of motion exercises were initiated for the patient after seven days. The patient did not develop any wound complications, and the stitches were removed on the second postoperative week. After surgery, the patient was given a shoulder sling, and medical oncology and radiation oncology were consulted. Medical oncology did not consider chemotherapy. Considering the wound’s condition, radiation oncology recommended a total dose of 60 Gy adjuvant local radiotherapy in 30 fractions one month after the surgery. No infection or wound healing problem was observed in the patient’s follow-up appointment. The patient’s hypoglycemia attacks ceased after surgery, and the blood glucose level ranged between 85 and 95 mg/dL during the day. With effective surgery and radiotherapy, there was no trace of recurrence or hypoglycemia attacks at the three-year follow-up appointment.

## Discussion

As mentioned earlier, paraneoplastic secretion of IGFs was seen in diverse soft-tissue sarcomas causing hypoglycemic attacks. These factors may mimic insulin and lower the blood glucose level [5]. Gherbon et al. showed a low level of insulin, C-peptide, and IGF-1 and a high level of IGF-2, revealing an increased IGF-2/IGF-1 ratio and indicating that insulin is not produced endogenously because of low C- peptide levels. The main secreted substance is IGF-2, causing paraneoplastic hypoglycemia [6]. The IGF-2 levels were quantitatively determined by radioimmunoassay methods [4]. The high IGF-2 level may indicate an active sarcoma. In that case, the tumor could not be resected, so chemotherapy and radiotherapy with glucocorticoid therapy were administered to the patient [6]. In our case, there was no need for glucocorticoid therapy because an immediate surgery was performed and the entire mass was removed.

In this case, hypoglycemia attacks ceased after the surgery, revealing a positive correlation between an active rapidly growing sarcoma, the level of paraneoplastic secreted substance, and its effect on body metabolism as lowering blood glucose levels. Although IGF signaling has been shown in rhabdomyosarcoma, synovial sarcoma, leiomyosarcoma, Ewing’s sarcoma, and osteosarcoma [5], the paraneoplastic secretion of IGF-2 remains debatable. However, the importance of IGF signaling and IGF-2 secretion of a myxofibrosarcoma was accepted because, with the aid of these substances, targeted therapy and monoclonal antibody for IGF signaling were developed. Our patient was treated with radiotherapy after surgery, but a targeted therapy option will be possible if the signaling and secretion of IGF are further studied. Further investigation of the IGF signaling pathway will also pioneer neoadjuvant and adjuvant treatment in bulky masses.

After the surgery, adjuvant chemotherapy and radiotherapy are important in treating soft-tissue sarcomas, such as myxofibrosarcoma. Surgical resection is indispensable in treatment. Surgery has progressed in recent years from large tissue amputation to local tumor resection [2]. Thus, aggressive tissue losses have been minimized, and flap application techniques have gained importance [7,8]. Although the tumor is completely resected and removed entirely (en bloc), the patient should be monitored closely because of the risk of recurrence. In a retrospective study by McMillan et al., high-grade tumors and tumors larger than 5 cm were associated with tumor recurrence [7]. In our case, adjuvant radiotherapy at a total dose of 60 Gy was administered in 30 fractions to the surgery area after special calculations to prevent a recurrence. However, radiotherapy has some complications around the wounds. Therefore, targeted therapy for IGF signaling may resolve the hypoglycemia problem, decrease the amount and frequency of radiotherapy, and reduce its complications. Moreover, the minimal residual disease may also be cured after targeted therapy.

One of the study’s limitations is that the level of insulin, IGF-1, and IGF-2 were not measured quantitatively using either blood samples or radioimmunoassay. In further cases, the levels of insulin, IGF-1, and IGF-2 should be measured before and after surgery in addition to the blood glucose and potassium levels to determine the cause of hypoglycemia attacks and the paraneoplastic over-secreted substance.

In this case, there was no need for massive tissue losses and more aggressive interventions (e.g., scapulectomy, disarticulation, thoracotomy) because early surgical excision was performed without local and systemic invasion of the tumor. In this way, the patient’s survival and quality of life were improved. Because the tumor was high grade and larger than 5 cm, the patient was subjected to frequent clinical follow-ups every three months in the first year, every six months in the second year, and once a year following that. In addition, the result of a tru-cut biopsy is insufficient to distinguish between low-, medium-, and high-grade myxofibrosarcoma and the importance of clean surgical margins and en bloc resection. Moreover, early surgical intervention will enable surgeons to prevent hypoglycemic attacks after surgical removal of the total mass so that the patient’s internal medicine problems can be resolved without medication. The effect of IGF signaling and IGF-2 redundancy on paraneoplastic hypoglycemia should be a focus of targeted therapy in further investigations.

## Conclusions

For the diagnosis of myxofibrosarcoma, the biopsy may show myofibroblastic differentiation but not the grade of the tumor. Therefore, early surgical intervention with wide resection is needed as it resulted in high-grade myxofibrosarcoma in our case. Hypoglycemia attacks may be a sign of the presence of a malignant tumor and may provide information about recurrence and the aggressiveness of the tumoral mass both at the beginning and during the follow-up period. In future research, the radioimmunoassay approach, which was lacking in our case study, may be used to identify the paraneoplastic-released chemical triggering hypoglycemic crises.
